# Anticancer Activity of Ferulic Acid-Inorganic Nanohybrids Synthesized via Two Different Hybridization Routes, Reconstruction and Exfoliation-Reassembly

**DOI:** 10.1155/2013/421967

**Published:** 2013-12-25

**Authors:** Hyoung-Jun Kim, Kitae Ryu, Joo-Hee Kang, Ae-Jin Choi, Tae-il Kim, Jae-Min Oh

**Affiliations:** ^1^Department of Chemistry and Medical Chemistry, College of Science and Technology, Yonsei University, Wonju, Gangwon-do 220-710, Republic of Korea; ^2^Department of Biosystems and Biomaterials Science and Engineering, College of Agriculture and Life Sciences, Seoul National University, Seoul 151-921, Republic of Korea; ^3^R&I Korea, GBU SC, Solvay, Industry-University Cooperation Building, Ewha Womans University, 150 Bukahyun-ro, Seodaemun-gu, Seoul 120-750, Republic of Korea; ^4^National Institute of Horticultural & Herbal Science (NIHHS) of RDA, Eumseong-gun, Chungcheongbuk-do 369-873, Republic of Korea

## Abstract

We have successfully prepared nanohybrids of biofunctional ferulic acid and layered double hydroxide nanomaterials through reconstruction and exfoliation-reassembly routes. From X-ray diffraction and infrared spectroscopy, both nanohybrids were determined to incorporate ferulic acid molecules in anionic form. Micrsocopic results showed that the nanohybrids had average particle size of 150 nm with plate-like morphology. As the two nanohybridization routes involved crystal disorder and random stacking of layers, the nanohybrids showed slight alteration in *z*-axis crystallinity and particle size. The zeta potential values of pristine and nanohybrids in deionized water were determined to be positive, while those in cell culture media shifted to negative values. According to the *in vitro* anticancer activity test on human cervical cancer HeLa cells, it was revealed that nanohybrids showed twice anticancer activity compared with ferulic acid itself. Therefore we could conclude that the nanohybrids of ferulic acid and layered double hydroxide had cellular delivery property of intercalated molecules on cancer cell lines.

## 1. Introduction

Organic-inorganic nanohybrids in which functional organic molecules were homogeneously combined with inorganic materials in nanoscale have attracted great interests in many research and industry fields [1, 2]. Among them the intercalation of biologically functionalized molecules into biocompatible 2-dimensional inorganics has been extensively studied during last decades. 2D layered materials, such as layered double hydroxides (LDHs), are known to have physical strength [3], chemical inertness [4], highly anisotropic structure [5], biocompatibility [6], pH-dependent solubility [7], and high cellular uptake efficiency [8] and therefore the intercalation of biofunctional molecules into LDHs has been known to enhance their biological availability [8, 9].

LDHs, of which general chemical formula is M(II)_1−*x*_M(III)_*x*_(OH)_2_(A^*n*−^)_*x*/*n*_·*m*H_2_O (M: metal, A: anionic species, *m* and *n*: integer, 0 < *x* < 1), have electrostatically stacked layers with interlayer anions [10, 11]. When a part of M(II) cations in M(II)(OH)_2_ structure is isomorphically substituted with M(III) ones, there evolves permanent positive charge on the framework. In this way, the layer of LDHs acquires positive layer charge which is compensated by the anions. Various kinds of biomolecules such as deoxyribonucleotides, oligonucleotides, anticancer drugs, antibiotics, vitamins and antioxidants have been incorporated to LDHs for various biological applications [8, 12, 13]. In this study, we intercalated ferulic acid (FA), a kind of antioxidant, into MgAl-LDHs in order to enhance the intrinsic anticancer activity of FA by increasing cellular uptake.

FA is one of phytochemical phenolic acids and widely known for its anti-inflammatory, antiviral immunoprotective, and antioxidant property [14–16]. FA molecules can inhibit cancer development by either scavenging reactive oxygen species or being involved in cell cycle upon cellular uptake [17], or they show antiproliferative and antimetastasis effect by specifically inhibiting antiapoptotic protein like Bcl-XL and Bcl2 [18]. The induction of detoxification by FA, specifically through the phase II metabolism, is also reported [17]. In spite of those advantageous properties, instability against oxidation, low solubility in water, and small cellular uptake limit the clinical applications [19]. Thus, the nanohybridization of phenolic acids or flavonoids with LDHs can be suggested as a strategy to enhance their biological functionality through stabilizing them from external stress, improving water solubility and increasing cellular internalization.

Generally, four kinds of reactions are utilized in the intercalation of functional organic molecules into LDH layers: coprecipitation, ion-exchange, reconstruction and exfoliation-reassembly. In coprecipitation, LDH's ionic precursors and organic molecules are dissolved in a solution and they are simultaneously precipitated into nanohybrids upon base titration [20]. On the other hand, the ion-exchange utilizes the already prepared LDH pristine and anionic organic molecules topotactically replace the preexisting one according to concentration gradient [21]. In reconstruction, the pristine LDHs with carbonate anion are first thermally treated at mild temperature (~400°C), and the resulting metal oxide (often referred to as layered double oxide (LDO)) recovers LDH structure under the existence of water and intended anions [22]. For exfoliation-reassembly, the LDH layers are first delaminated into a few sheets by treating in an appropriate solvent, and the obtained nanosheets are mixed with organic anions to restack the LDH structure [23]. The latter two methods are relatively less utilized conventionally but have advantages; the hybridization reaction occurs fast and the intercalation of large sized molecules is possible.

The intercalation of functional organic molecules into LDHs for biomedical applications has been extensively studied in various fields. Nevertheless, there have been not enough researches to investigate the effect of different intercalation methods on the physicochemical properties of organic-LDH hybrids and their biological activity. In this paper, we intercalated FA molecules into LDHs via reconstruction (REC) and exfoliation-reassembly (ER) route. The physico-chemical properties of resulting nanohybrids were characterized in detail compared with pristine LDHs. And the anticancer efficacy of nanohybrids was evaluated in the human cervical adenocarcinoma epithelial cell line, HeLa.

## 2. Materials and Methods

### 2.1. Materials

The magnesium nitrate hexahydrate (Mg(NO_3_)_2_·6H_2_O), aluminum nitrate nonahydrate (Al(NO_3_)_3_·9H_2_O), sodium bicarbonate (NaHCO_3_), dimethylsulfoxide (DMSO), thiazolyl blue tetrazolium bromide (MTT) and ferulic acid (C_10_H_10_O_4_) were purchased from sigma-aldrich Co., Ltd. (USA). Sodium hydroxide pellet (NaOH), formamide (CH_3_NO), and sodium nitrate (NaNO_3_) were obtained from Daejung Chemicals & Metals Co., Ltd. (Gyonggi-do, Korea). Dulbecco's Modified eagles' Medium (1X) + GlutaMax-1 (DMEM), Dulbecco's Phosphate Buffered Saline (DPBS), and 0.25% trypsin-EDTA were gained from Invitrogen-Gibco (Carlsbad, CA). All reagents were used without purification.

### 2.2. Preparation of the Pristine LDHs and Nanohybrids

For the preparation of CO_3_-LDH (Mg_2_Al(OH)_6_(CO_3_)_0.5_) pristine, the mixed metal solution (0.3 M of Mg(NO_3_)_2_·6H_2_O and 0.15 M Al(NO_3_)_3_·9H_2_O) was titrated with alkaline solution (0.9 M of NaOH and 0.675 M NaHCO_3_) until pH ~ 9.5. NO_3_-LDH pristine (Mg_2_Al(OH)_6_(NO_3_)) was synthesized by titrating mixed metal solution (0.3 M of Mg(NO_3_)_2_·6H_2_O and 0.15 M Al(NO_3_)_3_·9H_2_O) with alkaline solution (0.9 M of NaOH and supersaturated NaNO_3_) until pH ~ 9.5 with N_2_ gas purging. Both mixtures were hydrothermally treated at 150°C for 24 h. The products were freeze-dried after centrifugation and washing with decarbonated water.

In order to obtain FA-LDH (FL) nanohybrid via REC route (REC-FL), CO_3_-LDH was first calcined at 400°C for 8 h and mixed with FA solution with FA/Al ratio of 3 and then aged for 24 h at dark. The obtained precipitates were centrifuged, washed with decarbonated water, and then freeze-dried. To synthesize FL nanohybrid through ER (ER-FL), NO_3_-LDH was dispersed into formamide (0.5 g/L) and stirred for 24 h in order to delaminate LDHs as previously reported [21]. Thus obtained colloidal suspension was mixed with 0.07 M FA solution and then aged for 24 h at dark. Final products were collected, washed with decarbonated water several times, and freeze-dried.

### 2.3. Characterization

Powder X-ray diffraction (XRD) patterns were obtained with a Bruker AXS D2 Phaser with Ni-filtered Cu-K*α* radiation (*λ* = 1.5418 Å). Scans were from 3° to 80° with rate 0.02°/1 sec. The Fourier transform infrared (FT-IR) spectroscopy was obtained using Perkin Elmer, spectrum one B.v5.0 spectrometer with conventional KBr method. The scanning electron microscope (SEM) images were obtained with Quanta 250 FEG after Pt/Pd sputtering. The shape and thickness of pristine LDHs and nanohybrids were investigated using atomic force microscope (AFM) from NX10 (Park systems) with tapping mode at room temperature. For microscopies, all the samples were dispersed in deionized water (DW), gently deposited on a silicon wafer, and dried in ambient air. All the AFM images and profiles were recorded with 0.5 MHz scan rate and obtained images were analyzed using XEI software. Zeta-potential of pristine and nanohybrids in either DW or DMEM were measured by ELSZ-1000 (Otsuka). The hydrodynamic size of pristine and nanohybrids in DW were obtained with dynamic light scattering (DLS) mode of ELSZ-1000. The samples were prepared in 1 mg/mL concentration, and 0.5% Tween 80 (Daejung) was used as dispersant.

### 2.4. *In Vitro* Anticancer Activity Test

The anticancer activities of pristine LDHs, nanohybrids, and FA only were evaluated using the MTT assay with human cervical adenocalcinoma epithelial cells line (HeLa). Cells were cultured in DMEM (10% FBS) at 37°C with 5% CO_2_. The 100 *μ*L cultured HeLa cells (1 × 10^4^ cell/well) were seeded in a 96-well plate. After 24 h of incubation, the DMEM was removed and cells were treated with different concentrations of samples (FA and FL hybrid in serum-free DMEM (5% DMSO), concentration of FA to be 1, 5, 10, 20, 30, 40, and 50 *μ*M, and pristine LDH in serum-free DMEM (5% DMSO)). The dose of FL nanohybrids was determined to have equal amount with FA only and that of pristine was decided referring to FL nanohybrids. The cells were incubated for 24 h, and 25 *μ*L of MTT (2 mg/mL in DPBS) solution was added to each well and incubated for 2 h. The unreacted MTT was removed and formazan crystals formed by proliferating cells were dissolved in 150 *μ*L DMSO. The absorbance was measured using microplate reader (Synergy H1, BioTek, USA) at 570 nm.

## 3. Result and Discussion

**Figure 1:**
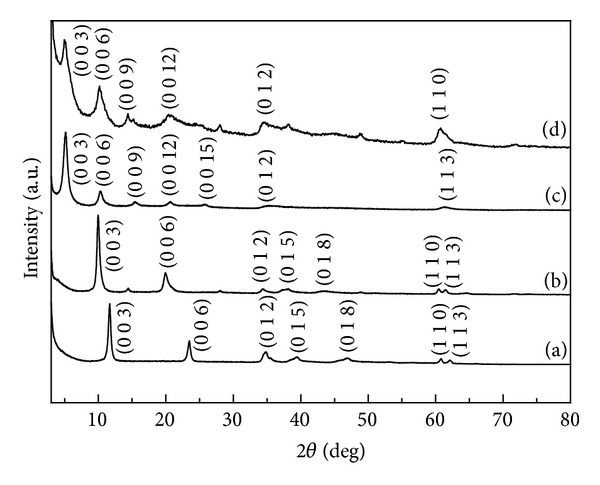
Powder X-ray diffraction patterns for pristine LDHs: (a) CO_3_-LDH, (b) NO_3_-LDH and nanohybrids, (c) REC-FL, and (d) ER-FL.

The powder XRD patterns of pristine and nanohybrids were displayed in Figure 1. The (hkl) index was identified based on JCPDS powder diffraction file 14-0191 and previous report [24]. As the LDH was 2-dimensional layered inorganic material of which interlayer ions existed through electrostatic interaction with layers, the interlayer distance could be varied according to the size of interlayer anions. The interlayer distance values could be calculated from (00l) diffraction peaks considering the layer thickness of LDH ~ 0.48 nm and were determined to be 0.28, 0.41, 1.23, and 1.28 nm for CO_3_-LDH, NO_3_-LDH, REC-FL, and ER-FL, respectively (Table 1). As the molecular dimension of FA was ~1.01 nm which was larger than CO_3_^2−^ or NO_3_^−^, the interlayer distance of LDH significantly expanded upon the intercalation of FA. According to the previous reports on FL nanohybrids, the interlayer distance of 1.2~1.3 nm stood for the zig-zag orientation of FA molecules enhancing their interlayer stabilization via *π*-*π* interaction of aromatic groups [13, 25].

The crystallite sizes of pristine and nanohybrids along *z*-axis were calculated by solving Scherrer's equation (equation below, where *λ*, *B*, and *θ* stood for wavelength of applied X-ray, full width half maximum of each peak, and the radian value of peak position) of (00l) peaks:

(1) $$\begin{array}{c} t=\frac{0.9\lambda }{B\cos\theta } \end{array}$$

The crystallite sizes of pristine LDHs were determined to be 18.6 and 14.5 nm for CO_3_-LDH and NO_3_-LDH, while nanohybrids showed relatively lower values of 12.4 and 10.4 nm for REC-FL and ER-FL, respectively (Table 1). This decrease in crystallinity along *z*-axis could be explained by the chemical change during nanohybridization process. In the reconstruction (REC) process, the pristine CO_3_-LDH first underwent heat treatment where sequential dehydration, dehydroxylation, and decarbonation occurred. The resulting metal oxide (LDO) could then be reconstructed into LDH phase in the presence of water and appropriate anionic species [22, 26]. During this retrotopotactic transformation, LDH went through a collapse in layered structure, resulting in the reduction of crystallinity in the layer stacking direction (*z*-axis) [27–29]. Similarly, exfoliation-reassembly (ER) process went with crystal disorder. The delaminated layers spontaneously restacked with anions forming house-of-cards-like structure with low crystallinity [30, 31]. The same reduced crystallinity and disorder of stacking in the nanohybrids were also visualized with SEM (Figure 3) and AFM (Figure 4), and will be further discussed in detail.

It is worthy to note here that the (hkl) peaks of pristine and nanohybrids were observed at similar 2-theta values, at around 35, 40, and 62 degrees for (012), (015), and (113), respectively. This showed that the crystallinity upon *xy*-plane direction was not significantly modified during nanohybridization. The calculated lattice parameters *a* and *c* for pristine and nanohybrids were displayed in Table 1. The *a* values lay between 0.303 and 0.305 nm, while *c* values showed clear variation. This result verified that the FA molecules were successfully intercalated into LDH interlayer without altering the 2-dimensional layer structure of LDH nanomaterials.

**Figure 2:**
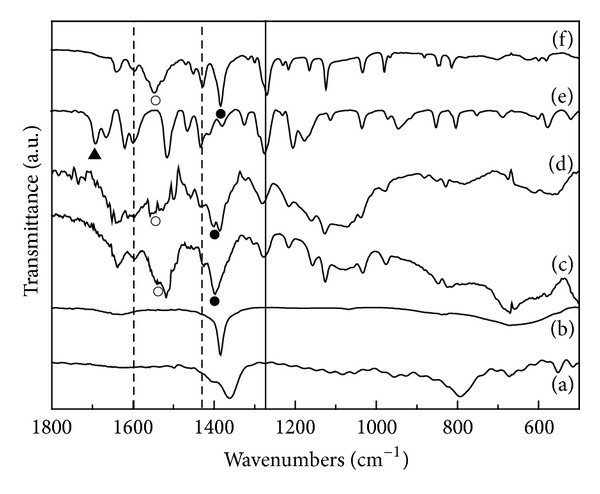
Fourier transform infrared spectra for pristine LDHs: (a) CO_3_-LDH, (b) NO_3_-LDH and nanohybrids, (c) REC-FL, and (d) ER-FL. FT-IR spectrum of (e) ferulic acid and (f) sodium ferulate salt is displayed for comparison.

The successful intercalation of FA moiety into LDHs was also verified with infrared spectroscopy. As shown in Figure 2, the FT-IR spectra of two nanohybrids, REC-FL and ER-FL, exhibited characteristic peaks of FA. Peaks at 1595 and 1428 cm^−1^ indicated with the dotted line in Figure 2 were attributed to the aromatic nucleus in FA. The peak at 1269 cm^−1^ marked with solid line was from the *ν*_as_(COC) stretching vibration. It revealed that the intact structure of FA was well preserved after hybridization reaction. The stretching vibration of carboxylic acid at 1692 cm^−1^ in FA (▲) split into asymmetric (*ν*_as_(COO^−^), ○ in Figure 2) and symmetric (*ν*_s_(COO^−^), ● in Figure 2) stretching vibration at 1541 and 1384 cm^−1^, respectively, after hybridization. The IR spectra of Na^+^-ferulate salt also showed the same splitting. Therefore, the carboxylic acid terminal of FA was thought to be deprotonated and stabilized between LDH layers via strong electrostatic interaction [13, 32]. When interpreting the IR spectra of carboxylate group, the energy difference between symmetric and asymmetric stretching (Δ*ν*_a-s_) was often utilized to determine the geometry of carboxylate and its interaction with counterion. For example, the Δ*ν*_a-s_ value was the largest when the carboxylate was unidentate to cation and it decreased in ionic state or bidentate coordination [33, 34]. The calculated Δ*ν*_a-s_ values for Na^+^-ferulate, REC-FL and ER-FL were fairly similar with 161, 145, and 157 cm^−1^, revealing that the FA moiety was electrostatically stabilized like in ionic lattice.

The size and morphology of inorganic nanoparticles are very important when those materials were utilized as drug encapsulating and delivering carrier into intracellular system as those properties have been reported to strongly affect the interaction between nanomaterials and biosubstances. LDH nanoparticles with size between 100 and 300 nm were reported to be easily taken up by cells and retained efficiently compared to smaller or larger ones [35]. It was also suggested that the rod morphology LDHs could have different subcellular compartment compared to plate-like ones and enter into the nucleus [36]. According to recent report on nanoparticles-cell interaction, the surface roughness of nanoparticles played an important role in determining the cellular uptake pathway as well as amount [37].

**Figure 3:**
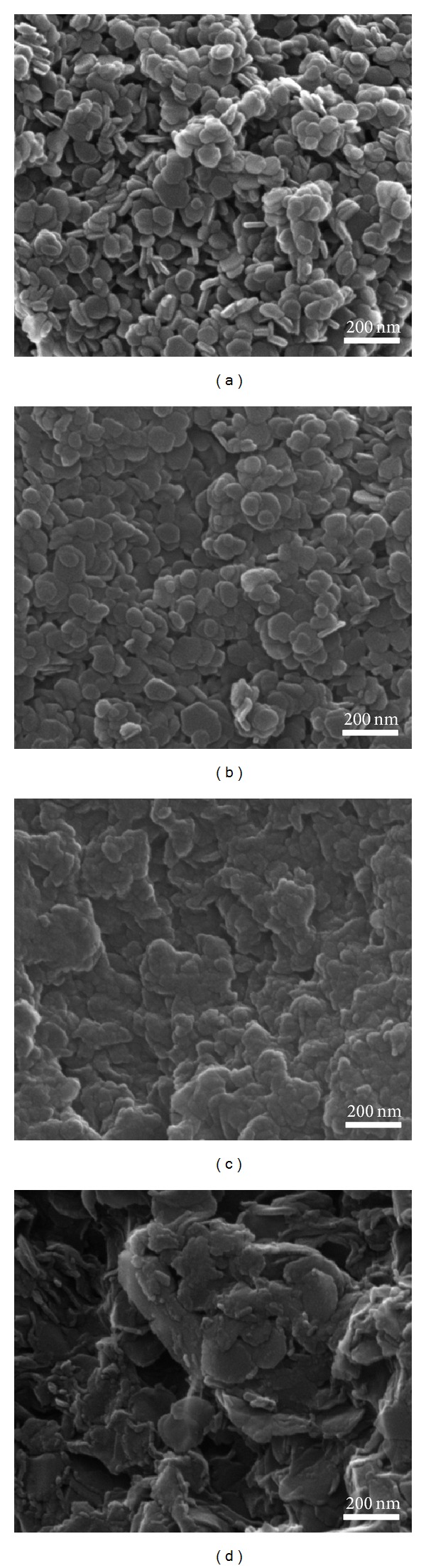
Scanning electron microscope images of pristine LDHs: (a) CO_3_-LDH, (b) NO_3_-LDH, and nanohybrids, (c) REC-FL, and (d) ER-FL.

**Figure 4:**
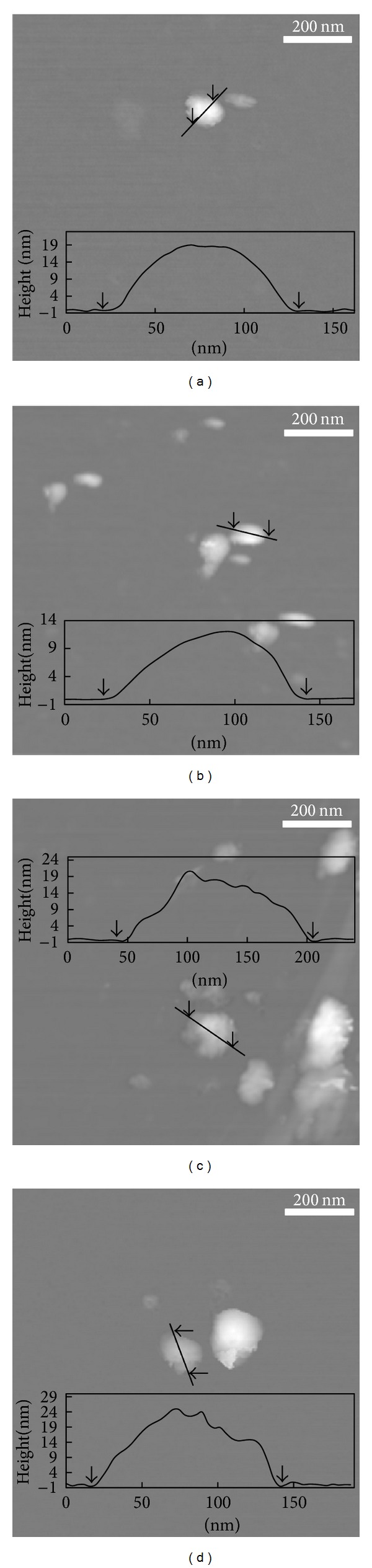
Atomic force microscopic images and height profile (inset) of pristine LDHs: (a) CO_3_-LDH, (b) NO_3_-LDH and nanohybrids, (c) REC-FL, and (d) ER-FL. The AFM images were obtained with tapping mode.

We evaluated both particle size and morphology of pristine LDHs as well as nanohybrids utilizing SEM and AFM. It was determined that all the samples had average primary particle diameter around 100 to 200 nm with homogeneous distribution and typical plate-like morphology (Figures 3 and 4). As previously mentioned, the hybridization methods, REC and ER, were different from the conventional ion-exchange reaction, which was a kind of topotactic reaction and the interlayer ions were simply exchanged with external ions through concentration gradient. However, the LDH lattices undergo chemical or physical treatment during REC and ER routes. Therefore, it is worthy to carefully evaluate the change in particle size and morphology between pristine and nanohybrids. The particle sizes calculated from randomly selected 100 particles of microscopic image were ~75, 145, 145 and 140 nm for CO_3_-LDH, NO_3_-LDH, REC-FL, and ER-FL, respectively, showing that the particle size of REC-FL slightly increased compared with its pristine CO_3_-LDH. It suggested that there were partial destruction and aggregation of particles during reconstruction process. Figure 4 showed that the overall crystallinity and smoothness of nanohybrids changed compared with pristine; the surface of nanohybrids was rugged and rough compared with the relatively clean surface of pristine LDHs. As discussed in the XRD patterns (Figure 1) and Scherrer's equation (Table 1), the LDH lattices underwent disorder of stacking during the REC or ER process. The particle thickness was determined to lie between 10 and 20 nm, suggesting that one particle, either pristine and nanohybrids, has tens of sheets-stacking structure.

Surface charge of nanomaterials was one of the major factors determining their interaction with cells. Nanomaterials, due to its very small size, had large portion of surface and therefore the surface properties took great part in determining their biological behavior. In particular, surface charge was the most important factor as the plasma membranes were known to have negative charge [38]. Many researchers have reported that the surface charge was strongly related to the biological behavior of nanomaterials such as plasma membrane interaction [39, 40], cellular uptake [41], blood-brain-barrier interaction [42], toxicity [43, 44], aggregation with proteins [45], and so forth. It was reported that the positively charged nanomaterials had active interaction with cellular membrane giving rise to enhanced cellular uptake. As LDHs had permanent positive layer charge, they have been reported to effectively deliver the intercalated molecules into intracellular system [46, 47].

**Figure 5:**
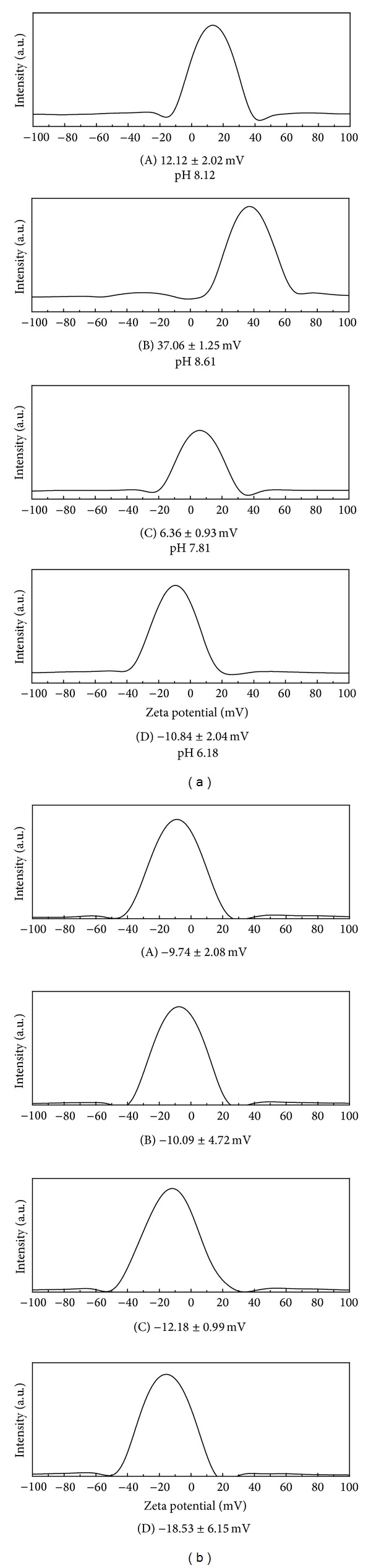
Zeta potential values of each sample in (a) deionized water and (b) DMEM media. Pristine LDHs: (A) CO_3_-LDH, (B) NO_3_-LDH and nanohybrids, (C) REC-FL, and (D) ER-FL.

Figure 5 showed the zeta potential of pristine LDHs and nanohybrids when they were dispersed in deionized water (DW) and cell culture medium, DMEM, respectively. The pH values of DW dispersion were also displayed in Figure 5(a). As the pristine LDHs had abundant hydroxyl groups on the surface which deprotonated surrounding water molecules, the pH of dispersion was slightly basic and the zeta potential was highly positive. On the other hand, nanohybrids suspensions (DW) showed relatively lower pH values and their zeta potentials slightly positive or negative values, possibly due to the adsorbed FA moiety on the rough surface of nanohybrids. Different from the DW dispersion, all the samples showed negative zeta potential values in DMEM environment. Cell culture media DMEM contained various anionic molecules like amino acids, chlorides, carbonates, phosphates, sulfates, and so forth, and they could reduce the positive zeta potential of LDHs by adsorbing on the surface. Similar zeta potential decrease from highly positive to negative direction in LDHs attributed to anionic species was reported previously [48, 49].

**Figure 6:**
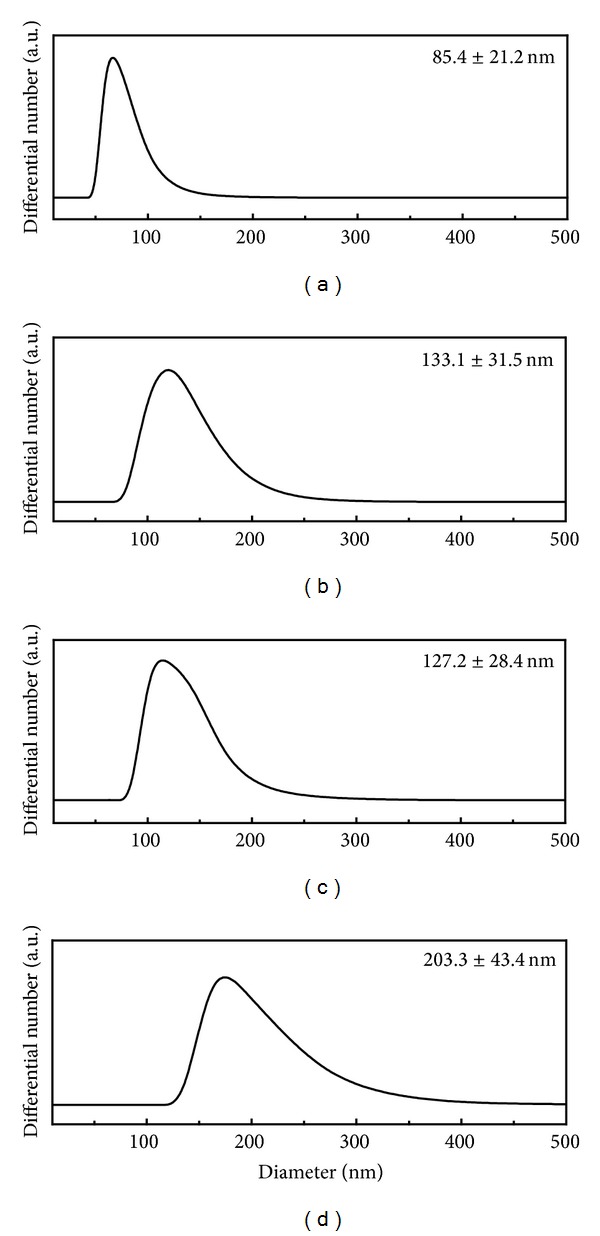
Hydrodynamic sizes of each sample in deionized water. Pristine LDHs: (a) CO_3_-LDH, (b) NO_3_-LDH and nanohybrids, (c) REC-FL, and (d) ER-FL.

We also evaluated the hydrodynamic size of pristine LDHs and nanohybrids in DW dispersion utilizing DLS. As shown in Figure 6, the pristine LDHs showed average diameter of 80~150 nm and nanohybrids exhibited slightly higher values of 100 to 200 nm, which well correspond to the results of microscopies (Figures 3 and 4). FA molecules on the surface of nanohybrids played as a kind of binder resulting in the slight aggregation in suspension. Nevertheless, the current nanohybrids were thought to show good cellular uptake property as the particle size of LDH below 300 nm was known to be effectively internalized into cells via endocytosis.

**Figure 7:**
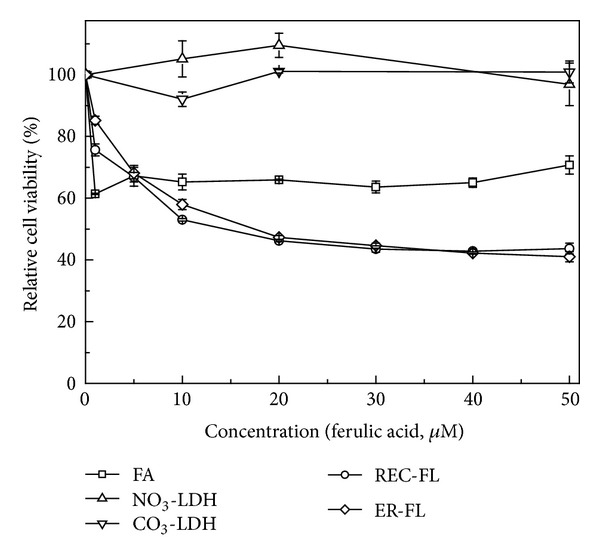
Concentration dependent anticancer activity of ferulic acid and nanohybrids, RE-FL and ER-FL, in HeLa cell lines.

Therefore, we evaluated the anticancer effect of nanohybrids in cultured cell line.  Figure 7 displayed the concentration dependent anticancer efficacy of FL nanohybrids compared with pristine LDHs and FA itself when they are treated to cervical cancer HeLa cells. For precise comparison, the dose of FL nanohybrids was set to have equivalent amount of FA, considering the chemical formula of nanohybrids. The FA only treated HeLa cells showed ~30% of cell viability decrease at the administration concentration 5 *μ*M and did not exhibit additional effect upon increasing concentration. On the other hand, both nanohybrids showed concentration dependent cell viability suppression upto ~60% until 50 *μ*M. Taking into account that the pristine LDH did not have cytotoxic effect regardless of concentration, the anticancer activity of nanohybrids almost twice of FA only was considered as the efficient cellular delivery ability of LDHs. In the previous research, it was revealed that the cellular uptake of anionic drug, methotrexate, was enhanced more than 10 times by intercalation into LDHs, which modified the cellular uptake pathway of drug molecules [47]. Similarly, we could suggest, in this study, that the nanohybridization of FAs with LDHs modified the cellular internalization route and increased the biological activity of FAs such as detoxification, inhibition of antiapoptotic proteins, and so forth. The two nanohybrids, REC-FL and ER-FL, showed almost similar anticancer efficacy although their crystallinity decreased compared with pristine. We could thus conclude that the crystallinity was not a critical factor in LDH nanohybrids in determining their cellular uptake. Both nanohybrids had good anticancer activity in spite of negative zeta potentials in DMEM dispersion. Even though the zeta potential was negative, the intrinsic positive charge of LDHs layer was thought to maximize interaction between nanohybrids and cellular membrane, increasing their cellular uptake.

## 4. Conclusion

We have demonstrated the physico-chemical property and biological activity of FA-LDH nanohybrids. Two different hybridization routes, reconstruction and exfoliation-reassembly, were utilized to intercalate FA molecules into LDH interlayer space. From XRD patterns and FT-IR spectra, it was revealed that the FA molecules were successfully stabilized between LDH layers through electrostatic interaction, although the crystallinity was slightly altered during intercalation route. Microscopic and DLS results revealed that the nanohybrids had less than 300 nm particle size which was an optimum value for cellular uptake. The anticancer efficacy of both nanohybrids showed concentration dependent manner with maximum twice higher cancer proliferation suppression effect compared to FA only. It was therefore concluded that the nanohybrids of FA with LDHs obtained by reconstruction or exfoliation-reassembly had beneficial physico-chemical properties for cellular uptake increasing the anticancer activity of the FA itself.

## Figures and Tables

**Table 1:** Crystallographic information of pristine LDHs and nanohybrids.

Sample	Interlayer distance (nm)	Crystallite size along *z*-axis (nm)	Lattice parameter	
			*a* (nm)	*c* (nm)
CO_3_-LDH	0.28	18.6	0.305	2.27
NO_3_-LDH	0.41	14.5	0.306	2.67
REC-FL	1.23	12.4	0.303	5.16
ER-FL	1.28	10.4	0.305	5.32

## References

[1] bin Hussein MZ, Zainal Z, Yahaya AH, Aziz ABA (2002). Synthesis of layered organic-inorganic nanohybrid material: an organic dye, naphthol blue black in magnesium-aluminum layered double hydroxide inorganic lamella. *Materials Science and Engineering B*.

[2] Oh J-M, Biswick TT, Choy J-H (2009). Layered nanomaterials for green materials. *Journal of Materials Chemistry*.

[3] Chen W, Qu B (2004). LLDPE/ZnAl LDH-exfoliated nanocomposites: effects of nanolayers on thermal and mechanical properties. *Journal of Materials Chemistry*.

[4] Choy J-H, Choi S-J, Oh J-M, Park T (2007). Clay minerals and layered double hydroxides for novel biological applications. *Applied Clay Science*.

[5] Wang X, Lu J, Shi W (2010). A thermochromic thin film based on host—guest interactions in a layered double hydroxide. *Langmuir*.

[6] Choi S-J, Oh J-M, Choy J-H (2009). Toxicological effects of inorganic nanoparticles on human lung cancer A549 cells. *Journal of Inorganic Biochemistry*.

[7] Choy J-H, Park M, Oh J-M (2006). Bio-nanohybrids based on layered double hydroxide. *Current Nanoscience*.

[8] Oh J-M, Park M, Kim S-T, Jung J-Y, Kang Y-G, Choy J-H (2006). Efficient delivery of anticancer drug MTX through MTX-LDH nanohybrid system. *Journal of Physics and Chemistry of Solids*.

[9] Lima E, Flores J, Cruz AS, Leyva-Gómez G, Krötzsch E (2013). Controlled release of ferulic acid from a hybrid hydrotalcite and its application as an antioxidant for human fibroblasts. *Microporous and Mesoporous Materials*.

[10] Kim TH, Kim HJ, Oh JM (2012). Interlayer structure of bioactive molecule, 2-aminoethanesulfonate, intercalated into calcium-containing layered double hydroxides. *Journal of Nanomaterials*.

[11] Abdolmohammad-Zadeh H, Tavarid K, Talleb Z (2012). Determination of iodate in food, environmental, and biological samples after solid-phase extraction with Ni-Al-Zr ternary layered double hydroxide as a nanosorbent. *The Scientific World Journal*.

[12] Oh JM, Choy JH, Choi SJ (2006). Nanoceramics-biomolecular conjugates for gene and drug delivery. *Advances in Science and Technology*.

[13] Biswick T, Park D-H, Shul Y-G, Hwang S-J, Choy J-H (2011). UV screening of ferulic acid-zinc basic salt nanohybrid with controlled release rate. *Journal of Nanoscience and Nanotechnology*.

[14] Kampa M, Alexaki V-I, Notas G (2004). Antiproliferative and apoptotic effects of selective phenolic acids on T47D human breast cancer cells: potential mechanisms of action. *Breast Cancer Research*.

[15] King PJ, Ma G, Miao W (1999). Structure-activity relationships: analogues of the dicaffeoylquinic and dicaffeoyltartaric acids as potent inhibitors of human immunodeficiency virus type 1 integrase and replication. *Journal of Medicinal Chemistry*.

[16] Lin L-C, Kuo Y-C, Chou C-J (1999). Immunomodulatory principles of Dichrocephala bicolor. *Journal of Natural Products*.

[17] Manson MM, Ball HWL, Barrett MC (1997). Mechanism of action of dietary chemoprotective agents in rat liver: induction of phase I and II drug metabolizing enzymes and aflatoxin B1 metabolism. *Carcinogenesis*.

[18] Karthikeyan S, Kanimozhi G, Prasad NR, Mahalakshmi R (2011). Radiosensitizing effect of ferulic acid on human cervical carcinoma cells in vitro. *Toxicology in Vitro*.

[19] Kikugawa M, Tsuchiyama M, Kai K, Sakamoto T (2012). Synthesis of highly water-soluble feruloyl diglycerols by esterification of an Aspergillus niger feruloyl esterase. *Applied Microbiology and Biotechnology*.

[20] Oh JM, Park DH, Choi SJ, Choy JH (2012). LDH nanocontainers as bio-reservoirs and drug delivery carriers. *Recent Patents on Nanotechnology*.

[21] Tezuka S, Chitrakar R, Sonoda A, Ooi K, Tomida T (2004). Studies on selective adsorbents for oxo-anions. Nitrate ion-exchange properties of layered double hydroxides with different metal atoms. *Green Chemistry*.

[22] Rives V (2001). *Layered Double Hydroxides: Present and Future*.

[23] Park D-H, Kim J-E, Oh J-M, Shul Y-G, Choy J-H (2010). DNA core@Inorganic shell. *Journal of the American Chemical Society*.

[24] Rossi C, Schoubben A, Ricci M (2005). Intercalation of the radical scavenger ferulic acid in hydrotalcite-like anionic clays. *International Journal of Pharmaceutics*.

[25] Ambrogi V, Fardella G, Grandolini G, Perioli L, Tiralti MC (2002). Intercalation compounds of hydrotalcite-like anionic clays with anti-inflammatory agents, II: uptake of diclofenac for a controlled release formulation. *AAPS PharmSciTech*.

[26] Pan D, Zhang H, Zhang T, Duan X (2010). A novel organic-inorganic microhybrids containing anticancer agent doxifluridine and layered double hydroxides: structure and controlled release properties. *Chemical Engineering Science*.

[27] Costa DG, Rocha AB, Souza WF, Chiaro SSX, Leitão AA (2012). Ab initio study of reaction pathways related to initial steps of thermal decomposition of the layered double hydroxide compounds. *The Journal of Physical Chemistry C*.

[28] Pérez-Ramírez J, Abelló S, van der Pers NM (2007). Memory effect of activated Mg-Al hydrotalcite: in situ XRD studies during decomposition and gas-phase reconstruction. *Chemistry—A European Journal*.

[29] Yang W, Kim Y, Liu PKT, Sahimi M, Tsotsis TT (2002). A study by in situ techniques of the thermal evolution of the structure of a Mg-Al-CO_3_ layered double hydroxide. *Chemical Engineering Science*.

[30] Paek S-M, Jung H, Lee Y-J, Park M, Hwang S-J, Choy J-H (2006). Exfoliation and reassembling route to mesoporous titania nanohybrids. *Chemistry of Materials*.

[31] Vial S, Prevot V, Leroux F, Forano C (2008). Immobilization of urease in ZnAl layered double hydroxides by soft chemistry routes. *Microporous and Mesoporous Materials*.

[32] Wang J, Cao Y, Sun B, Wang C (2011). Characterisation of inclusion complex of trans-ferulic acid and hydroxypropyl-*β*-cyclodextrin. *Food Chemistry*.

[33] Nara M, Torii H, Tasumi M (1996). Correlation between the vibrational frequencies of the carboxylate group and the types of its coordination to a metal ion: an ab initio molecular orbital study. *Journal of Physical Chemistry*.

[34] Yang J-H, Han Y-S, Park M, Park T, Hwang S-J, Choy J-H (2007). New inorganic-based drug delivery system of indole-3-acetic acid-layered metal hydroxide nanohybrids with controlled release rate. *Chemistry of Materials*.

[35] Oh J-M, Choi S-J, Lee G-E, Kim J-E, Choy J-H (2009). Inorganic metal hydroxide nanoparticles for targeted cellular uptake through clathrin-mediated endocytosis. *Chemistry—An Asian Journal*.

[36] Xu ZP, Niebert M, Porazik K (2008). Subcellular compartment targeting of layered double hydroxide nanoparticles. *Journal of Controlled Release*.

[37] Schrade A, Mailänder V, Ritz S, Landfester K, Ziener U (2012). Surface roughness and charge influence the uptake of nanoparticles: fluorescently labeled pickering-type versus surfactant-stabilized nanoparticles. *Macromolecular Bioscience*.

[38] Zhang Y, Yang M, Portney NG (2008). Zeta potential: a surface electrical characteristic to probe the interaction of nanoparticles with normal and cancer human breast epithelial cells. *Biomedical Microdevices*.

[39] Chen L, Mccrate JM, Lee JC-M, Li H (2011). The role of surface charge on the uptake and biocompatibility of hydroxyapatite nanoparticles with osteoblast cells. *Nanotechnology*.

[40] Su G, Zhou H, Mu Q (2012). Effective surface charge density determines the electrostatic attraction between nanoparticles and cells. *Journal of Physical Chemistry C*.

[41] Merhi M, Dombu CY, Brient A (2012). Study of serum interaction with a cationic nanoparticle: implications for in vitro endocytosis, cytotoxicity and genotoxicity. *International Journal of Pharmaceutics*.

[42] Lockman PR, Koziara JM, Mumper RJ, Allen DD (2004). Nanoparticle surface charges alter blood-brain barrier integrity and permeability. *Journal of Drug Targeting*.

[43] Chertok B, David AE, Yang VC (2010). Polyethyleneimine-modified iron oxide nanoparticles for brain tumor drug delivery using magnetic targeting and intra-carotid administration. *Biomaterials*.

[44] Marquis BJ, Liu Z, Braun KL, Haynes CL (2011). Investigation of noble metal nanoparticle *ζ*-potential effects on single-cell exocytosis function in vitro with carbon-fiber microelectrode amperometry. *Analyst*.

[45] Kendall M, Ding P, Kendall K (2011). Particle and nanoparticle interactions with fibrinogen: the importance of aggregation in nanotoxicology. *Nanotoxicology*.

[46] Choy JH, Kwak SY, Jeong YJ, Park JS (2000). Inorganic layered double hydroxides as nonviral vectors. *Angewandte Chemie—International Edition*.

[47] Oh J-M, Choi S-J, Kim S-T, Choy J-H (2006). Cellular uptake mechanism of an inorganic nanovehicle and its drug conjugates: enhanced efficacy due to clathrin-mediated endocytosis. *Bioconjugate Chemistry*.

[48] Pavan PC, Crepaldi EL, Valim JB (2000). Sorption of anionic surfactants on layered double hydroxides. *Journal of Colloid and Interface Science*.

[49] Xu ZP, Jin Y, Liu S, Hao ZP, Lu GQ (2008). Surface charging of layered double hydroxides during dynamic interactions of anions at the interfaces. *Journal of Colloid and Interface Science*.

